# The mitochondrial genome of *Eurycantha calcarata* Lucas, 1869 (Phasmatodea: Lonchodinae) and its phylogeny

**DOI:** 10.1080/23802359.2021.1964403

**Published:** 2021-09-30

**Authors:** Ke-Ke Xu, Qing-Ping Chen, Jia-Yin Guan, Zi-Yi Zhang, Kenneth B. Storey, Dan-Na Yu, Jia-Yong Zhang

**Affiliations:** a College of Chemistry and Life Science, Zhejiang Normal University, Jinhua, PR China; b Department of Biology, Carleton University, Ottawa, Canada; c Key Lab of Wildlife Biotechnology, Conservation and Utilization of Zhejiang Province, Zhejiang Normal University, Jinhua, PR China

**Keywords:** Lonchodinae, mitogenome, phylogeny

## Abstract

The Lonchodinae (Phasmatodea: Phasmatidae) is rich in insect species with more than 330 species of 40 genera. The phylogenetic relationships within Lonchodinae have been under debate. We successfully sequenced the complete mitogenome of *Eurycantha calcarata* Lucas, 1869 (Phasmatodea: Lonchodinae) with a length of 16,280 bp, which had the same genes and gene arrangements as those of various published papers on stick insects. The whole mitogenome and control region of *E. calcarata* had a high AT content of 78.2 and 85.9%, respectively. All PCGs used ATN as the start codon, and most PCGs used TAA/TAG as the stop codons excluding COX2 (T), COX3 (TA), and ND5 (TA). To discuss the phylogeny of Lonchodinae, we reconstructed the phylogenetic relationships of 27 species of Phasmatodea including *E. calcarata* and two species of Embioptera used as outgroups. In BI and ML trees, the monophyly of Lonchodinae and Necrosciinae was well supported, whereas the monophyly of Clitumninae was not recovered. These results indicated that Lonchodinae was a sister clade to Phylliinae and *E. calcarata* was a sister clade to *Phraortes* genus.

The phylogenetic relationships of Phasmatodea have always been unstable at high levels, and the phylogeny of the species-rich Lonchodinae remains unresolved (Bradler and Buckley 2018). Bradler et al. (2014) showed that Lonchodinae had a close relationship with Necrosciinae using three nuclear genes (18S and 28S rDNA, histone 3) and three mitochondrial genes (COX2, 12S, and 16S rRNA), which was consistent with the results of Tihelka et al. (2020). Song et al. (2020) supported that Lonchodinae was a sister clade to Phylliinae based on mitogenomes, which was also the results of some other studies (Zhou et al. 2017; Forni et al. 2021). However, some authors failed to recover the monophyly of Lonchodinae (Kômoto et al. 2011). More mitochondrial genomes need to be sequenced to clarify the monophyly of Lonchodinae. Therefore, we sequenced the mitogenome of *Eurycantha calcarata* Lucas, 1869 (Phasmatodea: Lonchodinae) to further discuss the phylogenetic relationship of Lonchodinae within Phasmatodea.

The samples of *E*. *calcarata* (Samples No. XJNY20200721-XJNY20200723) were retrieved from an insect-pet market in China, the source area being New Britain Island, Papua New Guinea (5°44′ S 150°44′ E). The samples were deposited at the Animal Specimen Museum, College of Life Sciences and Chemistry, Zhejiang Normal University, China (http://sky.zjnu.edu.cn/, Zhang JY, zhang3599533@16.com) under the voucher number XJNY20200721-XJNY20200723). Total DNA was extracted from foreleg muscle by Ezup Column Animal Genomic DNA Purification Kit (Sangon Biotech Company, Shanghai, China) and stored as the same place of samples. Universal primers were used to amplify some fragments of the mitogenome according to the methods of Zhang et al. (2018) using Sanger Sequencing. Then, the remaining gaps were sequenced by utilizing species-specific primers according to previously obtained sequences.

The complete mitochondrial genome of *E. calcarata* (GenBank No. MW915467) was a typical circular DNA molecule with a length of 16,280 bp. The mitochondrial genome of *E. calcarata* had the same genes and gene arrangements as those previously published for other stick insect species with 37 genes, including 13 PCGs, 22 *tRNA* genes, and two *rRNA* genes. The whole mitogenome and the control region of *E. calcarata* had a high AT content of 78.2 and 85.9%, respectively. The content of A was higher than T, and the content of G was higher than C. Twenty-four copies of tandem repeat regions with lengths of 32 bp each as well as two tandem repeat regions of 19 bp were found in the control region. All PCGs used ATN (N represents A, G, C, or T) as the initiation codon. Typical termination codons (TAA/TAG) were found in most PCGs, except for three incomplete terminal codons: COX2 (T), COX3 (TA), and ND5 (TA). The *16S rRNA* gene was located between trnL1 and trnV with a length of 1293 bp, whereas the *12S rRNA* gene was located between trnV and the control region with a length of 763 bp. The AT content of *16S rRNA* and *12S rRNA* genes was 79.5 and 75.7%, respectively. The length of the 22 *tRNA* genes was ranged from 62 to 70 bp. There were 19 *tRNA* genes that could be folded into the common cloverleaf model, except for trnS1that lacked the dihydrouridine (DHC) arm, trnR that lost the DHC loop, and trnN that lacked the TΨC loop.

To discuss the phylogenetic relationships of Phasmatodea, 29 species including *E. calcarata* were used; 28 mitochondrial genomes were downloaded from NCBI, including 26 species of Phasmatodea used as ingroups and two species of Embioptera used as outgroups (Cameron et al. 2006; Kômoto et al. 2011; Plazzi et al. 2011; Tomita et al. 2011; Kômoto et al. 2012; Song et al. 2016; Mikheyev et al., 2017; Zhou et al. 2017; Song et al. 2020; Dong et al. 2021). We aligned each of 13 protein-coding genes using Clustal W in the program Mega version 7. 0 (Kumar et al. 2016) and the program Gblock 0. 91b was used to identify conserved regions (Castresana 2000). The resulting alignments were concatenated with Geneious version 8.1.6 (Kearse et al. 2012). The phylogenetic relationship was constructed based on the 13 PCGs of the 29 species using Bayesian inference (BI) and maximum-likelihood (ML) methods *via* the programs MrBayes version 3.1.2 (Huelsenbeck and Ronquist 2001) and RAxML version 8.2.0 (Stamatakis 2014). In ML and BI analyses (Figure 1), Timematinae was located at the base of Phasmatodea, and the internal relationships of clades I–III showed identical topology. The monophyly of Lonchodinae and Necrosciinae was supported, whereas the monophyly of Clitumninae failed to recover. All phylogenetic results indicated that Lonchodinae was a sister clade to Phylliinae, which is consistent with other studies (Song et al. 2020; Forni et al. 2021). *E*. *calcarata* was a sister clade to *Phraortes* genus, then the clade of (*E. calcarata* + *Phraortes*) was a sister clade to *Megalophasma granulatum*.

## Data Availability

The genome sequence data that support the findings of this study are openly available in GenBank of NCBI (at https://www.ncbi.nlm.nih.gov/nuccore/MW915467) under the accession no MW915467. Phylogenetic relationships of Phasmatodea inferred from ML analysis (A) and BI analysis (B). The relationships of the phylogenetic tree among 27 species of Phasmatodea including *E. calcarata* (MW915467) used ingroups and two species of Embioptera used outgroups based on the nucleotide dataset of the 13 mitochondrial protein-coding genes. The phylogenetic relationships within clade I, clade II and clade III showed the same topology in BI and ML trees, respectively. The clade I included three species of Phylliinae and six species of Lonchodinae, the clade II included five species of Necrosciinae, and the clade III included the two species of Bacillinae, one species of Megacraniinae, one species of Extatosomatinae, one species of Phasmatinae, and five species of Clitumninae. Numbers around the nodes are the posterior probabilities of BI (right) and the bootstrap values of ML (left). The GenBank accession numbers of all species are shown in the figure. Black squares at nodes of the ML analysis indicate posterior probability less than 50 and black triangle at nodes of the BI analysis indicate bootstrap value less than 0.5.
